# Accuracy of *ICD-10* Diagnostic Codes for Identifying Housing Instability

**DOI:** 10.1001/jamanetworkopen.2024.25919

**Published:** 2024-08-05

**Authors:** Matthew J. O’Brien, Andrew Owen, Sharon Langshur, Bruce Doblin, Keiki Hinami, William Trick, David T. Liss

**Affiliations:** 1 Institute of Public Health and Medicine, Northwestern University Feinberg School of Medicine, Chicago, Illinois; 2 Division of General Internal Medicine and Geriatrics, Department of Medicine, Northwestern University Feinberg School of Medicine, Chicago, Illinois; 3 Health Research and Solutions Unit, Center for Health Equity and Innovation, Cook County Health, Chicago, Illinois

## Abstract

This cohort study assesses the performance of *International Statistical Classification of Diseases and Related Health Problems, Tenth Revision (ICD-10)* Z59 codes for identifying housing instability during health care encounters.

## Introduction

People experiencing housing instability, which comprises homelessness and other unstable housing circumstances, are at high risk for poor health outcomes.^1^ The *International Statistical Classification of Diseases and Related Health Problems, Tenth Revision (ICD-10)* includes Z59 diagnostic codes for housing instability, which are assigned by clinicians during health care encounters. Prior research suggests that Z59 codes are used infrequently.^2,3,4,5,6^ This study sought to evaluate the performance of Z59 codes for identifying housing instability during health care encounters using patient-reported data on housing instability as the reference standard to define this exposure.

## Methods

This retrospective cohort study included patients of any age from a Chicago-based Health Care for the Homeless Program (HCH) who had 1 or more ambulatory visit at the HCH where housing status was assessed and 1 or more encounter at another local health system that represented an opportunity to assign Z59 codes. The 6 other participating health systems contributed data from both ambulatory and hospital-based encounters. A central data hub merged and deduplicated the data to create a limited dataset across all health systems from January 1, 2016, to December 31, 2022. The study protocol was approved by the Chicago Area Institutional Review Board and deemed to be secondary data research for which consent is not required. We followed the STROBE reporting guideline.

Detailed data on housing status were collected by the HCH via patient questionnaires, which constituted the reference standard to ascertain housing instability. The second method for assessing housing instability, the presence of qualifying Z59 codes (homelessness [Z59.0x], inadequate housing [Z59.1, Z59.10, and Z59.19], or housing instability [Z59.81x]) documented during encounters at other participating health systems, was evaluated by calculating their performance characteristics.

Patients were categorized as ever experiencing housing instability according to HCH questionnaire data and qualifying Z59 codes from other participating health systems. We calculated the following performance characteristics of Z59 codes: sensitivity (proportion of patients experiencing housing instability who had a Z59 code), specificity (proportion not experiencing housing instability who did not have a Z59 code), positive predictive value (proportion with a Z59 code who were experiencing housing instability), and negative predictive value (proportion without a Z59 code who were not experiencing housing instability). Stata SE, version 18.0 was used to conduct statistical analyses.

## Results

Of 14 562 HCH patients who met inclusion criteria (37.9% female; 62.1% male; mean [SD] age, 43.8 [14.2] years), 4081 (28.0%) had housing instability and a Z59 code (group A), 78 (0.5%) did not have housing instability but had a Z59 code (group B), 10 369 (71.2%) had housing instability and no Z59 code (group C), and 34 (0.2%) did not have housing instability or a Z59 code (group D). Estimates of the Z59 code performance were as follows: sensitivity, 28.2%; specificity, 30.4%; positive predictive value, 98.1%; and negative predictive value, 0.3% (Figure).

**Figure.**
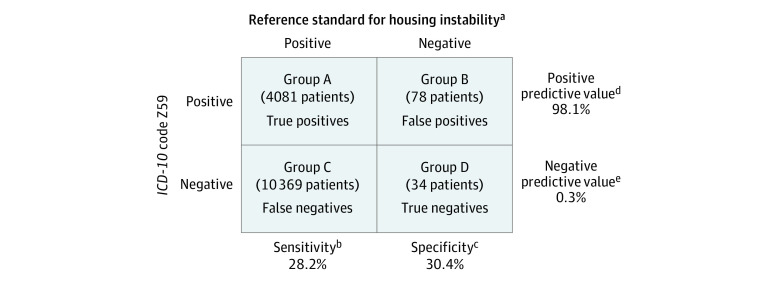
Performance Characteristics of *International Statistical Classification of Diseases and Related Health Problems, Tenth Revision (ICD-10)* Z59 Codes ^a^The reference standard for housing instability was provided by Healthcare for the Homeless program data. ^b^Sensitivity was calculated according to the following formula: A / (A + C). ^c^Specificity was calculated according to the following formula: D / (B + D). ^d^Positive predictive value was calculated according to the following formula: A / (A + B) ^e^Negative predictive value was calculated according to the following formula: D / (C + D).

## Discussion

To our knowledge, this is the first study examining the performance of Z59 codes that used patient-reported data as the reference standard to define housing instability. We found that Z59 codes exhibited poor overall performance, as evidenced by their low sensitivity and specificity. Because the negative predictive value was low, the absence of a Z59 code did not exclude housing instability. However, the high positive predictive value suggests that the presence of Z59 codes almost always indicates housing instability. A limitation is that we did not require a defined period between the HCH assessment of housing instability and other health care encounters when Z59 codes could be documented. However, most patients had a qualifying encounter within 180 days of completing the HCH housing questionnaire that was used to ascertain patients’ housing status. Additional research is needed to understand predictors of assigning Z59 codes during health care encounters for people experiencing housing instability. Interventions to increase screening and documentation of housing instability in health care settings are needed to enable the delivery of appropriate housing and medical services to this population.

## Notes

Supplement.
Data Sharing Supplement
