# Supplementary material for: Accuracy of ICD-10 Diagnostic Codes for Identifying Housing Instability
Source: JAMA Netw Open. 2024 Aug 5;7(8):e2425919. doi: 10.1001/jamanetworkopen.2024.25919 (PMC11301553; doi:10.1001/jamanetworkopen.2024.25919)
Supplement: Supplement. — Data Sharing Supplement [file jamanetwopen-e2425919-s001.pdf]

Data Sharing Statement

O'Brien. Accuracy of ICD-10 Diagnostic Codes for Identifying Housing Instability. *JAMA Netw Open*. Published August 05, 2024. doi:10.1001/jamanetworkopen.2024.25919

Data
Data available: Yes
Data types: Deidentified participant data
How to access data: Data sharing will be possible according to existing rules and regulations from the CAPriCORN research network.
When available: With publication

Supporting Documents
Document types: None

Additional Information
Who can access the data: Researchers whose proposed use of the data has been approved
Types of analyses: Depends on the approval for each data use
Mechanisms of data availability: after approval of a proposal with signed data agreement
Any additional restrictions: Data sharing must be approved by the CAPriCORN data network, and is under their sole discretion.
